# Extracellular Vesicles for Drug Delivery in Cancer Treatment

**DOI:** 10.1186/s12575-023-00220-3

**Published:** 2023-11-09

**Authors:** Li Wang, Xin Yu, Juan Zhou, Chunxia Su

**Affiliations:** 1 Department of Medical Oncology, Shanghai Pulmonary Hospital & Thoracic Cancer Institute, Tongji University School of Medicine, Shanghai, 200433 PR China; 2 Department of Clinical Research Center, Shanghai Pulmonary Hospital & Thoracic Cancer Institute, Tongji University School of Medicine, Shanghai, 200433 PR China; 3 Department of Medical Oncology and Clinical Research Center, Shanghai Pulmonary Hospital & Thoracic Cancer Institute, Tongji University School of Medicine, Shanghai, 200433 PR China

**Keywords:** Extracellular vesicles, Drug delivery, Cancer treatment, Natural compounds

## Abstract

Extracellular vesicles (EVs) are nanoscale vesicles derived from cells that mediate intercellular communication by transporting bioactive molecules. They play significant roles in various physiological and pathological conditions. EVs hold great potential as novel biomarkers of diseases, therapeutic agents, and drug delivery vehicles. Furthermore, EVs as novel drug delivery vehicles have demonstrated significant advantages in preclinical settings. In this review, we discussed the biogenesis and characteristics of EVs and their functions in cancer. We summarize the therapeutic applications of EVs as a natural delivery vehicles in cancer therapy. We highlight the existing challenges, illuminate vital questions, and propose recommendations to effectively address them effectively.

## Introduction

In the past decade, there has been rapid growth in our understanding of the types, characteristics, and physiological and pathological roles of extracellular vesicles (EVs). The International Society for Extracellular Vesicles (ISEV) recognizes EVs as general term for particles naturally released from cells, which are enveloped by a lipid bilayer and cannot replicate, and do not contain functional nucleus [1]. EVs can be broadly classified into three main subpopulations based on their biogenesis and size, including apoptotic bodies, microvesicles, exosomes and others [2–4]. EVs can be characterized based on their size and homogeneity (Table 1). Apoptotic bodies (1000—5000 nm) are the largest subpopulation of EVs released by cells undergoing apoptosis [5]. Microvesicles (also called ectosomes, 100 –1000 nm) are formed through the outward budding and fission of the plasma membrane and released into the extracellular space [6]. Exosome (40—160 nm) is the product of fusion between multivesicular bodies containing intraluminal vesicles and plasma membranes [7–10]. However, there is still a lack of confidence in identifying EV subtypes for a variety of reasons. In 2018, ISEV recommends using operational terminology to describe EVs subtypes when subcellular origin markers cannot be reliably established. This involves categorizing them based on their physical properties, biochemical composition, and describing their conditions or cellular origins. It is advised to avoid using historically vague, contradictory, and uncertainly generated terms like “exosome” and “microvesicle” [11].

**Table 1:** Subtypes of extracellular vesicles

**Subtype**	**Size**	**Markers**	**Biogenesis/release**	**Refs**
**Exosomes**				
**Exo-S**	50–70 nm	ESCRT complex proteins, CD9, CD63, CD81	Exo-S mainly contains proteins that associated with endosomes, multivesicular bodies, exosomes, and phagocytic vesicles. EXO-S is most likely a classical exosome	[9]
**Exo-L**	90–120 nm		Exo-L contains proteins that associated with composition of plasma membrane, cell–cell contacts or junctions, late endosomes, and trans-Golgi network. Exo-L may represent non-classical exosomes or extracellular vesicles from different subcellular origins	
**Microvesicles (Ectosomes)**	100 –1000 nm	Annexin A1, ARF6	Outward budding of the plasma membrane, scission/pinching off from membrane protrusions	[6]
**Apoptotic bodies**	1000—5000 nm	Phosphatidylserine	Released from apoptotic cells upon activation of apoptosis- related transduction pathways	[5]
**Exomere**	< 50 nm	Unknow	The biogenesis of exomeres may involve subcellular organelles or activities related to cellular metabolism, including organelles such as the endoplasmic reticulum and Golgi apparatus, as well as metabolic pathways within the cell	[9]
**Supermeres**	Unknown	Unknown	Unknown	[10]
**Exophers**	1,000–10,000 nm	Phosphatidylserine, LC3, Tom20	Unknown	[3]
**Retroviral- like particles**	Not determined	Gag- like proteins (Arc1, Arc2)	Unknown	[4]

EVs are secreted by both prokaryotic and eukaryotic cells. Most mammalian cell types, including neurons, endothelial cells, mesenchymal stem cells, and epithelial cells, have been shown to release EVs [11–16]. Furthermore, EVs have been detected in various biological fluids, including blood, urine, ascites, synovial fluid, and saliva [17–19]. It is noteworthy that certain subpopulations of EVs and particles have slightly different size ranges, biophysical characteristics, morphological characteristics, and protein marker expression, as well as the limitations of commonly used EVs purification methods, many studies have relied on analyzing mixed EVs populations consisting of exosomes, microvesicles, and non-membranous particles.

EVs play a critical role in intercellular communication and regulation, orchestrating diverse biological processes [20–24]. Internally, they harbor a repertoire of bioactive molecules including a blend of RNA, double-stranded DNA, proteins, lipids, glycoconjugates, and metabolites [25, 26]. This protective feature enables EVs to undergo long-range transfer across different tissues through blood circulation. Although the mechanisms underlying cargo sorting into EVs are not yet fully understood, it is evident that this process is highly selective [27]. Upon their release into the extracellular milieu, extracellular vesicles engage in intricate interactions with recipient cells, exerting profound influences on their functional capacities and physiological states. The levels of many cargo molecules in EVs do not directly correlate with their intracellular levels, indicating a regulated and specific sorting mechanism. Their multifaceted involvement spans both normal physiological dynamics and disease pathogenesis, endowing them with significant potential as biomarkers and promising tools for drug delivery and therapeutic interventions [28, 29].

Nowadays, cancer poses a formidable global threat to human life, driving researchers to seek novel effective anti-cancer therapeutics. In recent years, numerous drugs have showcased remarkable anti-cancer efficacy. However, the utilization of certain drugs is often hindered by intrinsic attributes such as limited target selectivity, short half-life in circulation, and unfavorable treatment-related side effects. EVs have emerged as highly advantageous vehicles for cancer treatment and drug delivery. In this review, we provide an in-depth exploration of the advancements in utilizing EVs for the translational study of cancer treatment, including their potential as innate delivery vehicles for anti-cancer drugs. Additionally, we discuss novel challenges within the realm of EV-based drug-loading strategies.

## Biogenesis of Extracellular Vesicle

EVs represent a heterogeneous population of membrane vesicles generated via diverse mechanisms. A growing body of evidence suggests that EVs play a key role in normal physiology as well as disease pathology. EVs are involved in the removal of unnecessary cellular components, mediating specific intercellular information exchange and communication, and activating intracellular signaling pathways [30]. By participating in cell-to-cell communication, EVs serve as homeostatic regulators in maintaining physiological and dynamic balance, as well as in the development and progression of diseases [31]. Moreover, EVs are also involved in various systemic pathological conditions, including blood coagulation, immune responses, infectious diseases, metabolic diseases, central nervous system-related diseases, musculoskeletal diseases, and cancer [32–34]. Discriminating EVs subtypes, defining their physiological relevance, regulating their production under pathological conditions, and harnessing them as therapeutic tools hold significant importance in the field.

Nowadays, the mechanisms underlying the generation, release, and uptake of EVs are relatively well understood. The generation of EVs typically relies on fundamental steps shared by various intracellular trafficking processes occurring in cellular compartments. These steps involve the formation of membrane microdomain enriched in specific cargoes and then budding and fission of the microdomain to generate vesicle. Several studies have demonstrated that various types of machinery regulate these steps [14, 15, 35–39], such as Rab family proteins, the ESCRT machinery, the syntenin-Alix pathway, tetraspanins, the cytoskeleton, and lipids. These sorting mechanisms selectively enrich specific cargoes into EVs, and their depletion can hinder the generation of specific EV subpopulations. The intracellular trafficking of recruiting cargoes between the plasma membrane and endosomes is a crucial regulatory factor governing the biogenesis of ectosomes and exosomes.

Extracellular factors including Microenvironmental pH, hypoxia, radiation, adhesion, and increased external pressure promote the release of EVs [34, 40–42]. EVs can influence adjacent and distant cells through autocrine or paracrine mechanisms [43]. Moreover, EVs can enter recipient cells through three main pathways: direct fusion with the cell membrane, receptor-ligand interaction, and fusion with the inner membrane after endocytosis [39]. The complex relationships between the generation, classification, and cargo of EVs still require continued investigation, as they can potentially give rise to distinct subpopulations of EVs, ultimately leading to potential physiological functions.

## Intercellular Communication of Extracellular Vesicle

EVs employ various mechanisms to mediate intercellular communication. The similarity of lipid membrane characteristics between EVs and the plasma membrane unveils a mechanism akin to phagocytic engulfment [44, 45], allowing for direct fusion with the recipient cell’s plasma membrane and facilitating the exchange of transmembrane proteins and lipids [46]. Moreover, EV uptake has been observed to transpire through a diverse array of well-established endocytic pathways and membrane fusion events, encompassing both grid protein-dependent and grid protein-independent mechanisms [47, 48]. The intrinsic heterogeneity exhibited by distinct subpopulations of EVs circulating in the organism poses a formidable challenge in unraveling the intricate physiological functional network governed by EVs-mediated intercellular communication. The precise capacity of EVs to selectively target specific cells and tissues remains an area of ongoing investigation. More and more studies are utilizing EV uptake mechanisms and cargo-loading strategies to develop novel therapeutic approaches.

## Sources of Drug Delivery

**Fig. 1:**
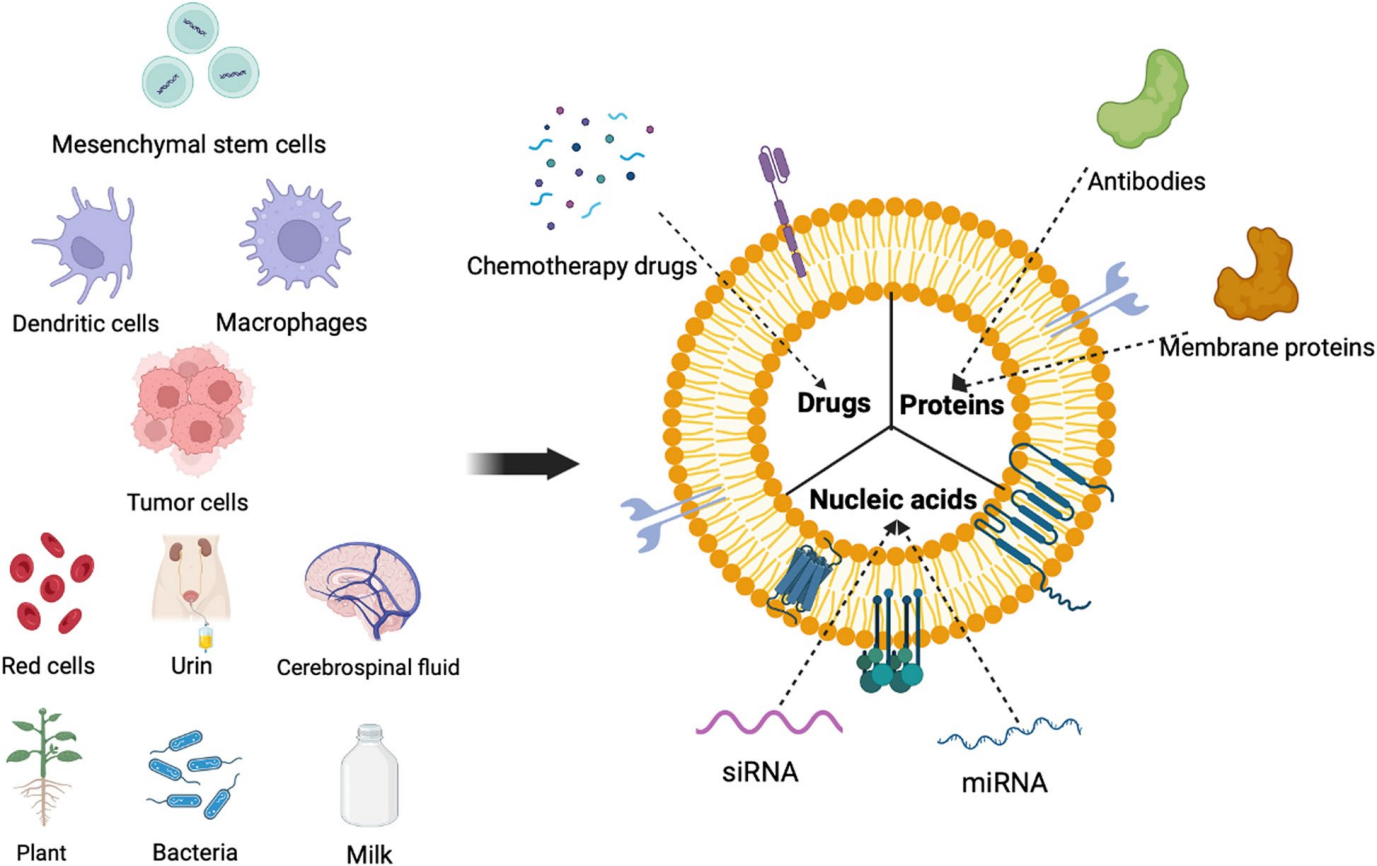
Extracellular vesicle-based drug delivery in cancer treatment

Drugs are limited in cancer treatment due to their poor targeting ability, poor bioavailability, unspecific cytotoxicity, and consequent systemic side effects [49]. There are many studies have demonstrated that EVs can serve as drug delivery vehicles, increasing drug accumulation in tumor tissues, extending blood circulation time, reducing systemic toxicity, and improving treatment efficacy (Fig. 1) [50–56].

### Nanotechnology Engineering

Recently, many studies have been utilizing nanotechnology engineering to enhance the efficacy of drug delivery. Lipid nanoparticles (LNPs) have garnered attention as nanocarriers for pharmaceuticals due to their size and ability to transport small molecules. LNP was approved by the FDA for encapsulating small molecules like doxorubicin and daunorubicin in order to treat cancer [57–59]. Cholesterol-conjugate loaded liposomes exhibited higher in vitro cytotoxicity compared to the use of 5-fluorouracil alone in the treatment of hepatocellular carcinoma [60]. Besides that, Niu et al. demonstrate a distinct design by patching doxorubicin-loaded heparin-based nanoparticles (DNs) onto the surface of natural grapefruit EVs, to fabricate biomimetic EV-DNs, achieving efficient drug delivery and thus significantly enhancing anti-glioma efficacy [61]. Although nanotechnology has various advantages in drug delivery vehicles, they are also accompanied by several drawbacks, such as high production costs and low drug loading capacity [62, 63]. These limitations have restricted further applications of nanotechnology.

### Extracellular Vesicles

#### Extracellular Vesicles Derived from Cells

Nowadays, EVs have advantages such as low toxicity, high biocompatibility, low immunogenicity, and inherent targeting ability, making them suitable as drug delivery carriers for cancer treatment. EVs that loaded with drugs derived from various cell sources, such as macrophages, dendritic cells, and red blood cells, have shown better anti-cancer effects [53, 64]. Therefore, selecting the most appropriate cell type for isolating EVs is crucial for drug delivery research.

##### Extracellular Vesicles Derived from Mesenchymal Stem Cells

Mesenchymal stem cells are widely used to produce EVs (MSC-EVs) due to their regenerative and immunomodulatory effects [65–68]. Pascucci et al. found that MSC-EVs containing paclitaxel can inhibit the proliferation of pancreatic cancer cells [54]. However, the application of mesenchymal stem cells in therapy has been limited due to their potential tumorigenicity.

##### Extracellular Vesicles Derived from Immune Cells

Another cell type that is widely used for EV isolation is immune cells, including macrophages, and dendritic cells [52, 64, 69]. Macrophage-derived exosomes loaded with paclitaxel have shown inhibitory effects in lung cancer metastasis mouse models. Furthermore, modifying these EVs with aminoethyl anhydride-polyethylene glycol carrier moieties is a common approach to reduce the immunogenicity of nanoscale particles. This modification improves the circulation time of EVs and enhances their lung-targeting capabilities for lung metastasis [51, 52]. EVs that derived from immature dendritic cells were engineered to express Lamp2b and fused with av integrin-specific iRGD targeting peptide. These modified exosomes were then loaded with doxorubicin using electroporation [53]. The presence of iRGD peptide enhanced the in vivo targeting of doxorubicin-loaded exosomes to MDA-MB-231 tumor cells, thereby enhancing their anti-tumor effects in established tumors. These findings suggest that immune cell-derived exosomes can be modified through various methods to enhance their targeting capabilities, making them more effective delivery vehicles.

##### Extracellular Vesicles Derived from Tumor Cells

Interestingly, EVs that are extracted from tumor cells are also utilized as a drug delivery vehicles. Due to the influence of the tumor microenvironment, cancer cell-derived EVs are generated abundantly and possess specific homing abilities [68, 70]. These EVs derived from cancer cells express tumor-specific antigens on their membranes, which may aid in generating anti-tumor immune responses in mouse models [71]. Notable outcomes were achieved in an investigation that focused on a mouse model of lung cancer. Compared with traditional chemotherapy, the administration of chemotherapy-loaded EVs resulted in a notable reduction in tumor burden and a prolongation of the survival period [70]. Moreover, a study showed that the utilization of platinum-loaded EVs derived from A549 cell line to treat three advanced lung cancer patients who exhibited resistance to platinum-based therapy. The results revealed a significant decrease in the number of tumor cells, while free platinum treatment failed to demonstrate beneficial effects for the patients. These collective findings underscore the considerable potential of cancer cell-derived EVs as a valuable approach in the treatment of lung cancer [70, 72]. Besides that, after loading with paclitaxel or gemcitabine, EVs derived from pancreatic cancer cells are employed for the treatment of pancreatic cancer. In vivo, pancreatic cell derived-EVs containing gemcitabine concentrate at the tumor site, leading to the inhibition of tumor growth while minimizing damage to normal tissues, significantly prolonging the survival rate of mice. This observation can be attributed to the potential tropism of EVs towards the tumor microenvironment, making EVs a competitive drug delivery vehicles for targeted chemotherapy [73]. However, cancer cell-derived EVs may promote tumor growth and metastasis by activating pathological pathways and exerting immunosuppressive effects. Subsequent research aimed to counteract this immunosuppressive response and found that when cancer cell-derived EVs were mixed with sufficient immune-stimulating adjuvants, the immunosuppressive effects were inhibited, thereby promoting anti-tumor responses [71].

##### Extracellular Vesicles Derived from Commonly Used Cellular Lines

In addition to the mentioned cells, there are other common cell lines that frequently used as sources of EVs for drug delivery, including Human Embryonic Kidney 293 cells (HEK293T), Chinese Hamster Ovary cells (CHO), and HeLa cells line. Among these, the HEK293T cell line is one of the most extensively utilized cell lines for EV-mediated drug delivery and has demonstrated potential value in industrial applications. While EVs derived from HEK293T cells can enrich certain cancer-related signaling molecules, they possess a higher transfection efficiency and are easily loaded with small therapeutic RNA molecules [74].

#### Extracellular Vesicles Derived from Body Fluids

Most body fluids contain EVs, including blood, urine, saliva, cerebrospinal fluid, ascites, and amniotic fluid, making these molecules useful for clinical diagnosis [75]. Among them, EVs derived from blood sources have been extensively studied as drug delivery vehicles [76, 77]. Usman et al. found that utilizing EVs derived from red blood cells can achieve highly efficient delivery of RNA, including small interfering RNA, antisense oligonucleotides, and CRISPR-associated protein 9 genome-editing guide RNA [77]. Zhang et al. showed that miR-155-loaded EVs from red blood cells exhibited excellent protective effects in acute liver failure, while EVs loaded with DOX or sorafenib showed significant therapeutic effects on in situ hepatocellular carcinoma without systemic toxicity [78]. In a clinical trial, Dai et al. showed EVs from ascites of colon cancer patients and combined them with granulocyte–macrophage colony-stimulating factor for immunotherapy. Combining these therapy strategy resulted in enhanced cytotoxic T lymphocyte responses specific to tumor antigens [79].

#### Extracellular Vesicles Derived from Other Sources

##### Milk

Recent research indicates that bovine milk-derived EVs have the potential value to serve as a drug delivery vehicles [80]. Bovine milk contains a higher abundance of separable EVs, and when injected into mouse models, bovine milk-derived EVs did not exhibit cytotoxicity or allergic reactions [81]. Importantly, bovine milk-derived EVs demonstrate high stability and low immunogenicity in the intestinal environment, making them potential carriers for chemotherapy drugs [82–84]. Furthermore, some studies have shown that bovine milk-derived EVs loaded with a range of drugs such as paclitaxel, docetaxel, and doxorubicin significantly improved the bioavailability and efficacy of these drugs in both in vitro and in vivo cancer models [85–87]. Therefore, researchers propose that drug-loaded milk EVs hold promise as a biocompatible, safe, effective, and cost-efficient targeted drug delivery mode for cancer treatment.

##### Plant

It has been discovered that plant-derived EVs for cancer treatment can be extracted from edible plants such as ginger, lemon, and grapefruit, which are non-toxic and are capable of being produced in large quantities [55, 88–91]. EVs derived from plants have established oral tolerance due to interactions with the intestinal immune system and food that we eat on a daily basis [92]. It has been reported that plant-derived EVs exhibit high resistance to gastric proteolytic enzymes and intestinal pancreas and bile extracts, making oral administration the most reasonable route, especially when targeting tumors located in the gastrointestinal tract [93]. Zhang et al. found that folate-modified and doxorubicin-loaded ginger-derived exosome-mimetic nanovesicles demonstrated excellent tissue compatibility and anti-tumor effects in colorectal cancer [55]. A study also demonstrated that grapefruit-derived nano-carriers effectively delivered various therapeutic drugs and enhanced their homing ability to inflammatory tumor tissues [90].

##### Bacteria

It has been demonstrated that bacterial outer membrane vesicles can carry immune stimulants and inducing the corresponding immune response can be used to treat tumors. Bacterial outer membrane vesicles derived from attenuated pneumococcal strains have been shown to effectively transport doxorubicin into A549 cells and interact with macrophages to activate the immune system, thereby enhancing the anti-tumor effect of doxorubicin [56]. It is worth noting that bacterial protoplasts, which lack toxic outer wall components, have become the preferred source of EVs. Kim et al. used Bacterial outer membrane vesicles derived from bacteria overexpressing epidermal growth factor to demonstrate the efficient delivery of doxorubicin and idarubicin to tumor cells in vitro and in vivo, resulting in the inhibition of tumor growth in mice [94].

## EVs as Drug Delivery Systems in Cancer Treatment

EVs have shown successful application in cancer therapy, facilitating the delivery of various therapeutic cargoes, including chemotherapy drugs, nucleic acids, and proteins [95, 96]. EVs are non-replicative and non-transformative, leading to fewer adverse reactions [97]. Moreover, the biodistribution of EVs varies based on the cell source, route of administration, and targeting methods [98, 99]. Various strategies of EVs have been investigated for tumor therapy (Table 2), and several ongoing clinical trials are investigating their potential as therapeutic delivery vehicles (Table 3) [72, 100–104].

**Table 2:** Different drug loading into EVs for cancer therapy

**Cargo**	**Years**	**Source of EVs**	**Cancer type**
**Doxorubicin**	2015	THP-1 macrophages	Ovarian and prostate cancer therapy
	2019	Pancreatic cancer cells, pancreatic stellate cells, and macrophages	Pancreatic cancer treatment
	2015	MCF-7 cells	Breast cancer therapy
	2019	MSC	Osteosarcoma therapy
	2013	Mouse immature dendritic cells (imDCs)	Breast cancer therapy
	2019	HEK293 cell	Breast cancer therapy
	2019	MDA-MB-231 cells	Cervical cancer therapy
**Paclitaxel**	2014	Murine SR4987 cells	Ductal pancreatic adenocarcinoma therapy
	2017	Bone marrow mesenchymal stromal cells (BM-MSCs)	Myeloma therapy
	2017	Canine mesenchymal stromal cells (cMSCs)	Glioblastoma treatment
	2016	RAW 264.7 macrophages	Lewis lung carcinoma therapy
	2017	Bovine milk	Lung cancer therapy
	2015	LNCaP and PC-3 PCa cell lines	Prostate cancer treatment
	2019	U-87 cells	Glioblastoma therapy
**Gemcitabine**	2020	Pancreatic cancer therapy	Pancreatic cancer therapy
	2020	M1 Macrophages	Chemoresistant pancreatic cancer treatment
	2020	Panc-1 cells	Pancreatic cancer therapy
**Paclitaxel, Doxorubicin and Gemcitabine**	2017	GinPa-MSCs	Oral squamous cell carcinoma therapy
**Paclitaxel and doxorubicin**	2015	Brain endothelial bEND.3 cells	Brain cancer therapy
	2020	RAW 264.7 macrophages	Triple negative breast cancer therapy
**Withaferin A, anthocyanidins, curcumin, paclitaxel and docetaxel**	2015	Bovine milk	Lung cancer therapy
**Methotrexate and cisplatin**	2012	A2780 human ovarian cancer cell	Hepatocarcinoma and ovarian cancer treatment
**Methotrexate**	2018	L929 cells	Glioblastoma treatment

**Table 3:** Clinical trials of extracellular vesicles as therapeutic delivery systems

**NCT number**	**Phase**	**Condition**	**Source of EVs**	**Cargo**
**NCT04592484**	Phase I/II	Advanced solid tumours	HEK293	STING agonist
**NCT03608631**	Phase I	Pancreatic cancer	Mesenchymal stromal cells	KRAS-G12D small interfering RNA
**NCT01294072**	Phase I	Colon cancer	Plant	Curcumin
**NCT01854866**	Phase II	Malignant pleural effusion	Tumour cells	Chemotherapy
**NCT02657460**	Phase II	Malignant pleural effusion	Tumour cells	Methotrexate
**NCT01159288**	Phase II	Non-small cell lung cancer	Dendritic cells	Peptides
**NCT01668849**	Phase I	Head and neck cancer	Plant	fentanyl patch
**NA**	Phase I	Melanoma	Autologous monocyte-derived dendritic cells	MAGE3
**NA**	Phase I	Lung cancer	A549	Cisplatin

### Chemotherapy Drugs

Doxorubicin and paclitaxel are commonly used carrier drugs for EVs drug delivery. However, their clinical application is limited due to dose-limiting toxicity and poor water solubility [105]. Additionally, their faces challenges in treating brain metastases due to its limited ability to cross the blood–brain barrier [106, 107]. And some studies have indicated that EVs can reduce the cardiac toxicity of doxorubicin by limiting its penetration into cardiac endothelial cells and accumulation in the heart [108, 109]. Furthermore, paclitaxel-loaded EVs have shown promising results in targeting and treating lung, breast, and pancreatic cancers [54, 64]. Besides that, Salarpour et al. demonstrated that EVs derived from U-87 MG cells can deliver paclitaxel across the blood–brain barrier, thereby improving the therapeutic effect of glioblastoma [110]. Barani et al. employed the film hydration method to develop novel niosomes containing cholesterol, Span, Tween, and gemcitabine. The efficacy of the niosomes was evaluated in vitro and in vivo. The developed niosomes show great potential as carriers for specific chemotherapeutic agents [111].

### Protein

Protein-based therapy is emerging as a promising approach for cancer treatment, addressing concerns related to maintaining activity and extending protein half-life. Various studies have explored the modification of EVs with specific proteins to enhance targeting abilities towards breast cancer cells in both in vitro and in vivo settings [112]. Koh et al. found that EVs with overexpressing SIRPα enhanced the phagocytosis of macrophages and effectively inhibited tumor growth in tumor-bearing mice [113]. Similarly, Hong et al. showed that EVs expressing PH20 hyaluronidase on the their surface can effectively degrade HA and induce DC activation through the TLR4 pathway, thereby inhibiting tumor growth [114]. Additionally, MSC-derived EVs with TRAIL overexpression such as cancer cell apoptosis in a dose-dependent manner [115].

Recently, a study has reported an innovative immunotherapy that uses EVs as targeted delivery vehicles for antibodies to breast cancer cells. By genetic modifications, exosomes are engineered to express two monoclonal antibodies, leading to a potent anti-tumor immune response by influencing T lymphocytes and breast cancer cells [116, 117]. Furthermore, researchers discovered that T-DM2, a promising drug for HER2-positive cancer, can be loaded into exosomes derived from HER2-positive cancer cells and delivered to other cancer cells via exosomes, resulting in apoptosis [118].

### Nucleic Acids

Currently, nucleic acids, such as miRNAs and siRNAs, hold promise as potential therapeutic strategies in cancer treatment [119]. Its clinical application is limited by its short half-life, immunogenicity, inability to penetrate physical barriers, and off-target effects. To address these challenges, EVs have gained significant attention as nucleic acid delivery vehicles, leveraging their unique properties to overcome these obstacles [120].

#### MicroRNAs

miRNAs, short non-coding RNAs involved in various cellular processes, play a significant part in cancer development and progression [121]. Some studies have showed that EVs derived from MSCs with deliver miRNAs to treat liver cancer by promoting apoptosis, and enhancing chemotherapy sensitivity in many cancers including breast, ovarian, pancreatic, and osteosarcoma [122–126]. Conversely, the delivery of miRNA inhibitors can also achieve anti-tumor effects, particularly when targeting miRNAs with tumor growth-promoting properties [127]. Enhancing the loading efficiency of miRNAs into EVs is an active area of research. For instance, Li et al. successfully enriched miR-155 in EVs by fusing the exosome surface marker protein CD9 with HuR, as demonstrated in their study [128]. Lang et al. found that EVs loaded with miR-124a significantly increased the median survival rate of glioblastoma mice [129]. These findings hold promise in advancing miRNA-based therapies using EVs as drug delivery vehicles.

#### Small Interfering RNAs

Delivering siRNA to target cells for gene silencing is a crucial gene therapy approach. Various studies have demonstrated the potential of EVs in efficiently loading and delivering siRNAs to tumor cells [130, 131]. To enhance delivery accuracy and efficiency, Pi and Zheng et al. modified EVs with RNA aptamers or folic acid in order to improve the efficiency and accuracy of siRNA delivery and uptake by prostate and breast cancer cells [132, 133]. Moreover, Kamerkar et al. targeted the delivery of siRNA-loaded engineered exosomes to pancreatic cancer cells, which showed significant tumor suppression both in vitro and in vivo, and this technology has been approved and entered clinical trials [101, 134].

## Encapsulation of Therapeutics

There are two main strategies for loading drugs into EVs: pre-loading and post-loading. Pre-loading involves loading the drug into the parent cells before EVs are isolated, resulting in EVs that carry the loaded drug. Post-loading, on the other hand, is the process of directly loading drugs into EVs after they have been separated, using passive or active methods.

### Pre-loading

Pre-loading facilitates the consistent and straightforward production of drug loaded EVs while preserving membrane integrity. Two prevalent methods of pre-loading include co-incubation and transfection.

Under specific conditions, the drugs are co-cultured with parent cells, facilitating their spontaneous absorption by cells through interaction with the lipid bilayer. The co-incubation method is employed to load various chemotherapeutic drugs, especially lipophilic ones like DOX and PTX [108, 135, 136]. Co-incubation is a relatively straightforward technique, but it is associated with lower loading efficiency. Additionally, its effectiveness is notably influenced by many factors inckuding drug properties, drug concentration gradients, and the specific type of parent cells [137]. Recent studies have indicated that Ultraviolet Induction cells can more efficiently load co-incubated drugs into EVs [72]. Furthermore, parent cells can overexpress therapeutic cargo and encapsulate cargo in EVs through cell transfection. ExoIL-12 loaded with PTGFRN is the world’s first engineered exosome candidate drug to enter clinical trials [138]. Cell transfection offers the advantages of high repeatability and simplicity. However, its drawbacks include low transfection efficiency and high dependence on cell viability [139, 140].

### Post-loading

Post-loading involves directly loading drugs into isolated EVs. Compared to pre-loading, this strategy is more customizable and minimizes interference from other substances. Currently, post-loading is mainly divided into passive loading and active loading methods.

When high concentrations of drugs are co-incubated with EVs, they passively diffuse into the lumens of EVs through interactions with the lipid bilayer. Passive loading method has been widely applied in cancer treatment [80]. However, the primary limitations of passive loading application are its low loading efficiency and limited selectivity.

Some cargo cannot passively diffuse through the EV membranes, physical induction or chemical induction are required to temporarily affect the permeability of EV membranes to enable the cargo entry. Physical induction typically involves instantaneous disruption of EV membranes by external forces. And chemical induction utilizes transfection agents to facilitate cargo loading without damaging EV membranes [141, 142]. Fuhrmann et al. found that saponin significantly increased the loading efficiency of porphyrins (derived from MDA-MB-231) into EVs [141]. Additionally, Zhang et al. developed an improved method for transfecting miRNAs into EVs using calcium chloride [143]. In comparison to electroporation, this approach exhibits comparable transfection efficiency with the added benefits of being simpler and more stable.

## Isolation and Purification of EVs

EVs are isolated from large volumes of conditioned media in industrial manufacturing. The primary contaminants include various vesicles, EV aggregates, cellular debris and organelles, DNA, cell necrosis products, free proteins, and protein aggregates. It is crucial to avoid microbial contaminants such as bacteria, fungi, and mycoplasma. Besides that, EVs are isolated from small quantities of highly complex biological samples in scientific research. These samples demonstrate notable diversity in their composition. The main contaminants are non-EV nanoparticles, primarily lipoproteins, ribonucleoproteins, and protein aggregates. Common indicators used to assess EVs purity include the particle-to-protein ratio and the protein-to-lipid ratio [144]. In summary, there are significant differences in the requirements and challenges for isolating EVs between industrial manufacturing and scientific research, primarily due to variations in sample volume, sample complexity, and the nature of contaminants. Customized isolation techniques are employed to ensure the purity and integrity of isolated EVs for their intended applications in these contexts. Currently, various methods have been developed to specifically isolate different subsets of EVs, such as centrifugation-based methods, precipitation-based methods, and others.

Ultracentrifugation (UC) is considered the gold standard of EV isolation in entrifugation-based techniques. UC employs centrifugal force to pellet EV particles, effectively separating them from major contaminants such as proteins and small molecular compounds, which remain in the supernatant. However, UC also has several drawbacks, including low EV yield, partial vesicle damage and aggregation, co-pelleting of non-exosomal components, and the formation of aggregated contaminants, primarily protein aggregates [145].

In precipitation-based methods, EVs can be reversibly aggregated after treatment with various chemical reagents, such as polyethylene glycol, precipitation with cationic polymers, and PROSPR approach [146, 147]. EVs can be separated through medium-speed centrifugation. These techniques are straightforward, relatively cost-effective, and do not require complex equipment. However, as precipitation methods lack selectivity towards EVs, the purity of the isolated EVs tends to be relatively low.

Other methods for isolating EVs include Size-based methods (ultrafiltration, tangential flow filtration, size-exclusion chromatography, asymmetrical flow-field-flow fractionation), chromatography methods (anion-exchange chromatography, hydrophobic chromatography), and affinity-based isolation methods. Table 4 summarizes the comparison of EV isolation methods.

**Table 4:** Comparison of EVs isolation approaches

**Isolation methods**	**Principle**	**Advantages**	**Limitations**
**Centrifugation-based methods**			
Ultracentrifugation	Centrifugal force	(1) Cost-effective; (2) No supplement required	(1) Low EV yield; (2) Disruption and aggregation of EVs; (3) Coisolation; (4) Time- and equipment-consuming; (5) Low reproducibility
Multiple-step centrifugation	EV isolation by sedimentation	(1) Well validated; (2) Suitable for a large volumeof sample; (3) No additional reagents required	(1) Efficiency varies among different biological Sources; (2) EV integrity may be compromised; (3) Time consuming; (4)Requires an expensive ultracentrifuge for small EV
Density gradient ultracentrifugation	EV isolation by size and density	(1) Efficient at preserving EV characteristics; (2) Suitable for downstream analysis; (3) High purity	(1)Time consuming; (2)Subjected to operator-based variability; (3)Low yield; (4)Requires expensive ultracentrifuge
**Precipitation-based methods**			
Precipitation with cationic polymers	Sedimentation of EVs using polymers and sedimentation	(1) Simple; (2) Not equipment- consuming; (3) High EV recovery	(1) Low purity; (2) Contamination with polymers and non-EV particles; (3) Low reproducibility
**Size-based methods**			
Ultrafiltration	Filtration through semi-permeable membrane	(1) Medium-to-high yield; (2) Simple; (3) scalable; (4) fast	(1) Possible contamination with proteins; (2) Non-specific binding of EVs to membrane; (3) Possible EV damage
Tangential flow filtration	Filtration through a semi-permeable membrane with tangential flow	(1) High yield; (2) Scalable; (3) High purity; (4) High reproducibility; (5) Optimal as pre-concentration method	(1) Special equipment required; (2) Contamination with large proteins and non-EV particles
Asymmetrical flow-field-flow fractionation	Separation in parabolic flow according to diffusion capacity	(1) High reproducibility; (2) Separation of heterogeneous fractions	(1) Special equipment required; (2) Limited scalability
**Chromatography methods**			
Anion-exchange chromatography	EV adsorption onto positively-charged sorbents	(1) High yield; (2) High purity; (3) High reproducibility	(1) Requires pre-concentration for large volumes; (2) Contamination with non-EV particles (3) Requires an additional buffer- exchange step
Hydrophobic chromatography	Adsorption of uncharged vesicles onto hydrophobic sorbent in high-salt buffer	(1) High purity; (2) Fast; (3) Low cost; (4) Scalable	(1) Variable EV yield
**Affinity-based isolation methods**			
Beads conjugated with antibodies against tetra- spanins	EV isolation via highly specific interactions with surface markers	(1) Highest purity; (2) High recovery; (3) Fast; (4) Not equipment-intensive; selective for EVs	(1) High cost; (2) Highly limited scalability for most approaches

## Engineering of EVs

Surface engineering is a significant milestone in the field of EVs formulations. Assessing the effectiveness of surface engineering is crucial for evaluating the therapeutic efficacy of EV formulations, especially in the context of precision medicine. Genetic methods involves modifying the cells responsible for EV production to express a fused genetic construct, which includes a fundamental EV protein linked to a targeting moiety associated with the targeting molecule, such as Lamp2b, CD9, CD63, and PTGFRN [148–150]. For example, HER2 is significantly overexpression in various tumors such as breast cancer, ovarian cancer, and gastric cancer. DARPins are a class of recombinant binding proteins that can bind to HER2 with high specificity. A study using lentiviral transduction of donor cells, successfully prepared hybrid EVs that expressing Lamp2b-DARPin G3 [130]. This achievement enables precise targeted delivery of TPD52 siRNA to HER2-positive breast cancer cells. Furthermore, PDGFR is a single-chain transmembrane glycoprotein that commonly employed to anchor fusion proteins. the PDGFR-GE11 peptide can specifically bind to EGFR, thereby targeting tumors with EGFR expression [151].

The fusion of the target molecule with the membrane protein to create a chimeric protein has the potential to modify the structure, integrity, and functionality of the native anchoring proteins on the surface of EVs. Many studies have indicated that the surface of EVs is enriched with negatively charged phosphatidylserines [152, 153], and lactadherin’s C1C2 domains specifically bind to phosphatidylserines. Kooijmans et al. utilized genetic engineering to equip EVs with a lactadherin-streptavidin fusion protein. They created C1C2-anti-EGFR recombinant fusion proteins, enabling precise targeting of tumor cells [154]. This engineering strategy holds promising potential as an effective system for cancer therapy. Furthermore, glycosylphosphatidylinositol-anchored proteins are also abundant on the surface of EVs. Various functional ligands, such as nanobodies, reporter proteins, and immune-stimulatory molecules, can bind to glycosylphosphatidylinositol and be expressed on the surface of EVs [155]. CD47 is a transmembrane protein that enriched on the surface of EVs, with its N-terminus positioned on the external surface of the EV. A study fused two different peptides (CDX or CREKA peptides) to the N-terminus of CD47, enabling specific targeting of U87 and GL261 glioblastoma cells, thus achieving a targeted effect on glioblastoma [156]. Besides that, a study engineered T cells to release EVs that carrying chimeric antigen receptors (CARs) with single-chain variable fragments of the antibodies cetuximab or trastuzumab [157]. These CAR-EVs demonstrated significant anti-tumor effects and exhibited low toxicity.

## The Challenges of Extracellular Vesicles as Drug Delivery

The main challenges of drug delivery include off-target toxicity within target tissues, rapid clearance, low bioavailability. There are many synthetic delivery vehicles have been developed. Liposomes are the most widely clinically approved carriers on the clinical. The advantages of EVs are clearly superior to liposomes in the field of drug delivery vehicles. EVs, originating from the organism itself, with low immunogenicity, thereby ensuring excellent tolerance and safety. Additionally, EVs have the capability to traverse the blood–brain barrier and enter the bloodstream within the brain [158]. This allows for the rapid delivery of drugs to lesions within the brain, offering a potential treatment option for intracranial diseases. EVs can undergo artificial surface modification to express specific molecules, thereby enhancing their targeting capabilities. Therefore, many studies have demonstrated that EVs as drug delivery vehicles are an ideal strategy for treating various types of cancer. However, there are several challenges that require clarification [159].

First, Optimal methods for loading drugs into EVs and quantification need a thorough evaluation. Some methods, like ultrasound, may be more efficient than others but could compromise the structural integrity of EVs [110]. Loading external chemical or biomolecules into EVs is a significant challenge. The composition of EVs introduces another issue, as it may lead to the transfer of undesirable content derived from the parent cells, potentially triggering immunogenic or oncogenic responses [160]. Rapid clearance of EVs and excessive immune system activation after administration are potential drawbacks of using EVs as drug delivery tools, choosing of administration route critical [159].

Secondly, EVs are inherently highly heterogeneous, and their functions and effects may vary depending on the carried cargo [161]. Therefore, careful selection of the appropriate EV subtype for specific drug delivery is essential [162]. EVs reflect their source cells and may carry substances that could unexpectedly promote cancer development during cancer treatment [163]. Additionally, EVs may exhibit significant differences in their biological distribution and half-life based on their cell of origin [164]. In the context of utilizing EVs as drug delivery systems, it’s crucial to assess potential interactions between the loaded exogenous cargo and endogenous cargo. This evaluation is pivotal in determining whether off-target effects [138]. Thus, comprehensive preclinical evaluations on cells, tissues, and animal models are crucial before considering their application.

Third, Other disadvantages of using EVs as drug delivery vehicles include challenges in their production, purification processes, and a lack of reproducibility in drug loading techniques [165]. The commercialization of EVs also encounters challenges including technical, economic, and regulatory issues [166].

Fourth, interdisciplinary collaboration plays a crucial role in the development of therapeutic strategies of EVs. Collaboration between researchers from fields such as cell biology, engineering, and immunology is instrumental in advancing more effective EV-based therapeutic approaches. It is critical to understanding of the generation and release mechanisms of EVs. The classification of EVs remains unclear. Recently discovered like exomeres and suprameres demand further investigation. Moreover, it is crucial to refine production processes or innovate new isolation techniques to augment both the yield and purity of EVs. This advancement will significantly broaden the spectrum of drug-loading options for EVs. Additionally, it is essential to investigation the interaction between EVs and the human immune system. This involves exploring strategies for surface engineering of EVs to further improve their targeted drug delivery capabilities while reducing their immunogenicity.

In summary, EVs as drug delivery requires utmost caution. One of the main advantages of employing EV-based drug delivery is their ability to reduce cytotoxicity. Therefore, drug-loaded EVs should demonstrate superior efficacy, tolerability, and safety in cancer treatment.

## Discussion

Nearly all cells release EVs as heterogeneous lipid nanoparticles. They participate in both proximal and distal cell communication [167]. EVs play a crucial role in regulating various physiological and pathophysiological processes. The majority of cell- and animal-based experimental evidence supports the significant role of EVs in almost all aspects of cancer, spanning from cancer initiation and progression to the development of paraneoplastic syndromes [168]. While several drugs have been developed to inhibit the biogenesis or secretion of tumor-derived EVs, they have not yet received clinical approval. Further research involving relevant preclinical human cancer models and clinical trials targeting the depletion of tumor EVs may contribute to the development of novel anticancer therapies.

EVs are a group of small lipid-based nanoparticles decorated with complex surface proteins and lipids, facilitating homing to specific tissues. The composition and biogenesis of EVs directly depend on their sources. These characteristics and their natural advantage makes EVs a useful vehicle for delivering therapeutic payloads due to their advantages as nanocarriers. Unlike artificially engineered nanoparticles, EVs occur naturally and, therefore, do not elicit inflammatory reactions. The tissue-homing ability of EVs allows them to reach distant target sites. This novel therapeutic strategy is currently under preclinical investigation for various cancer types, showing promising results with minimal side effects. In recent years, new approaches have been continuously developed to improve these nanovesicles, such as the development of superparamagnetic nanoparticles based on EVs [169–171]. However, a number of challenges must be overcome before drug-loaded EVs for cancer treatment can be commercialized. Therefore, further research and exploration of new strategies are required to enhance the production and drug-loading efficiency of EVs.

## Data Availability

Not applicable.
